# The Use of Nano-Hydroxyapatite (NH) for Socket Preservation: Communication of an Upcoming Multicenter Study with the Presentation of a Pilot Case Report

**DOI:** 10.3390/medicina59111978

**Published:** 2023-11-09

**Authors:** Roberto Rossi, Elisabetta Carli, Fabrizio Bambini, Stefano Mummolo, Caterina Licini, Lucia Memè

**Affiliations:** 1 Independent Researcher, 16149 Genova, Italy; drrossi@mac.com; 2 Unit of Pediatric Dentistry, Department of Surgical, Medical, Molecular and Critical Area Pathology, University of Pisa, 56126 Pisa, Italy; elisabettacarli1@gmail.com (E.C.); l.meme@staff.univpm.it (L.M.); 3 Department of Clinical Sciences and Stomatology, Polytechnic University of Marche, 60126 Ancona, Italy; 4 Department of Life, Health and Environmental Sciences, Università degli Studi dell’Aquila, 67100 L’Aquila, Italy; stefano.mummolo@univaq.it; 5 Department of Clinic and Molecular Science, Polytechnic University of Marche, 60126 Ancona, Italy; c.licini@staff.univpm.it

**Keywords:** biomaterial, nanohydroxyapatite, guided bone regeneration, socket preservation

## Abstract

***Background and Objectives***: The use of biomaterials in dentistry is extremely common. From a commercial perspective, different types of osteoconductive and osteoinductive biomaterials are available to clinicians. In the field of osteoconductive materials, clinicians have biomaterials made of heterologous bones at their disposal, including biomaterials of bovine, porcine, and equine origins, and biomaterials of natural origin, such as corals and hydroxyapatites. In recent years, it has become possible to synthesize nano-Ha and produce scaffolds using digital information. Although a large variety of biomaterials has been produced, there is no scientific evidence that proves their absolute indispensability in terms of the preservation of postextraction sites or in the execution of guided bone regeneration. While there is no scientific evidence showing that one material is better than another, there is evidence suggesting that several products have better in situ permanence. This article describes a preliminary study to evaluate the *histological results*, *ISQ values,* and *prevalence* of nano-HA. ***Materials and Methods***: In this study, we planned to use a new biomaterial based on nanohydroxyapatite for implantation at one postextraction site; the nano-HA in this study was NuvaBONE (Overmed, Buccinasco, Milano, Italy). This is a synthetic bone graft substitute that is based on nanostructured biomimetic hydroxyapatite for application in oral–maxillofacial surgery, orthopedics, traumatology, spine surgery, and neurosurgery. In our pilot case, a patient with a hopeless tooth underwent extraction, and the large defect remaining after the removal of the tooth was filled with nano-HA to restore the volume. Twelve months later, the patient was booked for implant surgery to replace the missing tooth. At the time of the surgery, a biopsy of the regenerated tissue was taken using a trephine of 4 mm in the inner side and 8 mm deep. ***Results***: The histological results of the biopsy showed abundant bone formation, high values of ISQ increasing from the insertion to the prosthetic phase, and a good reorganization of hydroxyapatite granules during resorption. The implant is in good function, and the replaced tooth shows good esthetics. ***Conclusions***: The good results of this pilot case indicate starting the next Multicentric study to have more and clearer information about this nanohydroxyapatite (NH) compared with control sites.

## 1. Introduction

The dental clinical practice often involves the extraction of hopeless teeth and their replacement with osseointegrated implants. If anatomical conditions make this possible, and the residual socket is suitable for inserting an implant, it is possible to perform extraction and implant at the same time. If there is not enough volume and apical bone after the extraction to achieve primary stability of the fixture, the clinician will have to perform a guided bone regeneration procedure to restore sufficient quantity and quality of the bone to host an implant. The GBR takes into consideration the use of different types of biomaterials. In particular, reabsorbable biomaterials, before being replaced by bone, are degraded due to the pH of the solution that surrounds them or the phagocytic/osteocytic activity of macrophages and osteoclasts. On the other hand, nonabsorbable biomaterials occupy a space that cannot be replaced by bone and are encapsulated in the bone matrix.

Based on their behavior toward bone cellular components, we can differentiate osteoconductive materials and osteoinductive materials [1].

With the exception of autologous bone, synthetic growth factors, and amelogenins, other materials on the market are all osteoconductive.

Osteoconduction is a biological characteristic common to all autologous bone substitutes, which, although occurring mostly in materials of bone origin, remains strongly conditioned by the bone activity of the recipient site [2]. After positioning the bone substitute, a clot forms around it, and endothelial, mesenchymal, and inflammatory cells arrive by crossing the walls of the recipient site. The grafted material then becomes a structure through which new bone tissue can begin to be generated.

The healing mechanism of these bone substitutes differs from that of autologous bone grafts; because of the absence of osteoinductive capacity, their healing mechanism delays, sometimes considerably, the times of neoangiogenesis and remodeling [3]. The inflammatory picture that forms in autologous grafts 7 days after surgery shows a continuous healing process, whereas the healing process of nonautologous grafts continues until the second week, with significantly slowed neoangiogenesis. The reduced vascular supply reduces the migration of new vital cells, and a picture of chronic inflammation begins to develop. If the immunological reaction, which is determined by the nonautologous material used, causes little or no immune response, it will undergo integration approximately 2 months after the operation. Therefore, there will be new bone formation at the periphery of the graft and the creation of a rich vascular network within the graft itself, with the speed of reabsorption and remodeling depending on the type of replacement material used [4].

However, if an immunological reaction occurs, the graft will undergo rejection, often after a latency period, which is characterized by the chronicity of the inflammatory process, the destruction of blood vessels, the formation of thrombi, and an increase in osteoclastic activity, with the consequent detachment of the graft from the recipient site [5]. Among the currently most used materials on the market, we find heterologous bone of animal origin, including bovine, equine, and porcine origins. Similar to autologous and homologous bone, it is available as spongy or cortical bone tissue. Among the available biomaterials made of heterologous bone, the most used are those made of bone of bovine origin [6,7,8], which have long been used in experimental and clinical experience, showing a reliable osteoconductive action and long remodeling and reabsorption times. The bone of bovine origin is deproteinized, deantigenized, and subsequently sterilized. Physically, it occurs in the form of spongiosa or cortical granules that are formed by aggregates of thin lamellae of 100 Å in thickness, 100 Å in length, and 400 Å in width, as well as in blocks. This material is made of bovine apatite, which, like human bone, consists of apatite crystals that are organized to form lattices with micro- and macropores, which favor both the stability of the clot and the addition of new bone within its structure. This material can be used alone or mixed with autologous bone in various percentages, thus combining its osteoinductive capacity and ability to maintain its volume over time with the osteogenic and inductive capacity of autologous bone. Inorganic bovine apatite integrates well into the recipient site and undergoes slow reabsorption. Recently, apatites of *bovine origin* [9] with a double porosity morphology have been introduced; these apatites are obtained by sintering calcium phosphate powder into granules with a size ranging from 250 to 600 μm. They have a bimodal porosity ranging from 10 to 60 μm, in which the large pores communicate with each other via the small pores. This morphology seems to promote the formation of an organic matrix within the porosity as if these granules are equipped with osteoinductive capacity and could attract biocomponents into circulation.

Materials of *equine origin* [10] undergo enzymatic deantigenation procedures and present a temporal reabsorption compatible with the normal turnover of human bone (approximately 12 months).

Materials of *porcine origin* [11], which still require deantigenation, have an average reabsorption of 12–24 months. We can find nonautologous biomaterials of biological origin that are derived from marine algae on the market and available to clinicians. Among these, we find corals [12], obtained from calcified marine algae and consisting mainly of calcium carbonate, in the form of porous aragonite (a metastable form of calcium carbonate in the crystalline phase, which has a three-dimensional structure similar to that of bone). These have a porosity greater than 45%, with pores with a diameter of approximately 150 μm. They come in the form of granules or blocks and have very long resorption times of up to 24 months after the grafting procedure, during which the bone grows around and within these granules.

Phylogenic hydroxyapatites [13] are also obtained from calcified marine algae via pyrolytic fractionation, with hydrothermal transformation of calcium carbonate.

They are formed by numerous channels with the interposition of 1 μm pores. The orientation of these channels allows the penetration of fluids and cells inside the biomaterial, thus significantly increasing the contact surface between biological fluids, and consequently, allowing faster colonization by macrophage cells.

These materials also have an excellent osteoinductive capacity and have a resorption time of 3 years. Synthetic bone substitutes deserve a separate discussion, as all of these biomaterials show that they possess osteoconductive capacity and maintain stable bonds with the newly formed bone over time. Morphologically, they can be porous, crystalline, amorphous, or granular. Among these biomaterials, we find *bioglass* [14] with a particular composition of a vitreous nature, which, when surgically inserted into the bone tissue, is integrated by the latter. Bioactive glasses are those that stimulate new osteogenesis. The osteoconductive capacity of the phosphate salts present in these materials has made them known in orthopedics since the 1960s. However, they also demonstrate their biocompatibility in preprosthetic implant surgery since they do not cause immunological reactions, allergies, inflammatory reactions, or side effects.

Bioglasses contain silica as a flux, sodium oxide as a stabilizer, calcium oxide, and, to a lesser extent, phosphorus pentoxide. Their bioactivity is linked to the surface hydrolytic degradation process of glassy phosphosilicate [15]. The course of the hydrolytic and biological process, which leads to the formation of a bond between bioactive glass and bone tissue, is linked to the surface reaction of the glass with blood plasma. This reaction is characterized by the migration of calcium, phosphorus, and silicon ions from the glass and the development of a layer of silica in the form of a gel in the glass–bone tissue interface, with a high content of hydroxyl ions. This layer constitutes an active site, which, due to the competition of phosphorus and calcium ions present in the glass and in the biological liquid, grows and develops into amorphous calcium phosphate, which will then transform into hydroxyapatite. This apatitic surface layer is recognized on its own by osteogenic cells, and a stable bond is thus established between the glass and the bone. Bioglass comes in the form of granules with a diameter of 300 μm. The distance that exists between the various particles that make up the bioglass ensures an optimal space for the infiltration and regeneration of bone tissue. Calcium and phosphate ions are released by the material and are reabsorbed by the body’s fluids to be used in bone formation. After positioning, the material undergoes a resorption process, which begins 2 to 16 weeks after surgery and is associated with replacement with osteoblasts, which deposit newly formed bone around the granules. This resorption process can last up to 16 months after surgery. According to some experimental studies, bioglasses have osteoinductive capabilities; thus, they are considered scaffolds that are capable of acting as a support for osteogenesis in the induction phase. *Polyglycolic and polylactic acid polymers* [16] are highly biocompatible synthetic products that do not induce immunological or inflammatory reactions, have osteoconductive capabilities, and can be completely replaced by trabecular bone.

These materials come in the form of blocks, granules, and gels, and as they are not radiopaque, they allow a better evaluation of the formation of bone tissue in the months following their application. Currently, the use of polymers in the form of polylactic and polyglycolic acid gels, in a 50:50 ratio, is implemented in association with other heterologous materials [17], which become more easily treatable due to the aggregating action of these gels. Among the available synthetic substitute materials are those with the fastest resorption, which generally occurs within 60–90 days. These polymers have been found to be very biocompatible, but, although no noteworthy contraindications have been reported, they have yet to be fully studied. Calcium sulfate is one of the best-known biomaterials, which has been used in orthopedics since 1900. Although its composition is very similar to that of hydroxyapatite, calcium sulfate is distinguished from the latter by its higher calcium content, its different density and solubility, its different chemical–physical properties, and, above all, its capacity to be completely reabsorbed without triggering inflammation or foreign body reaction.

*Calcium sulfate* [18] hemihydrate exists in two forms: alpha and beta. The beta form is the most commonly used and is prepared as granules or, more rarely, as a powder after dry sterilization. It is a material with a high osteoconductive capacity that is completely reabsorbed and replaced by osteoid tissue in a variable time between 6 and 20 months. The resorption processes begin from the sixth week after the graft procedure and the granules act as nuclei promoting new bone formation, which occurs starting from the surface of the granules. This material is often used mixed with autologous bone in preimplant bone reconstruction operations.

Finally, *hydroxyapatite* is another topic of our research [19,20].

Synthetic hydroxyapatite is one of the longest-standing and most studied synthetic materials on the market. This material represents the inorganic component of human bone and has a strong osteoconductive capacity. The various hydroxyapatites on the market are mostly synthesized as microgranules of various diameters ranging between 200 and 500 μm or alternatively in blocks. Their porosity varies from 70 to 90%, with pores that are connected to each other with spaces of 80 to 200 μm in between, which facilitates colonization by osteogenic cells. In these hydroxyapatites, the conformation of crystals is also important, as it is able to influence the processes of osteointegration by modulating the participation of physiological liquids, cells, and proteins. Their geometry also seems to constitute an ideal microenvironment to concentrate growth factors and stimulate neoangiogenesis. These materials are frequently used mixed with autologous bone to increase the volume of the graft, with an osteoconductive material that acts as a support. The resorption times of these materials are very long, up to 4 years, and are influenced by the size of the pores and granules.

Nanocrystalline hydroxyapatites have recently been introduced to the market, with granules that have a very large specific surface area, which have been developed with the aim of shortening resorption time. There are many aspects that are still unknown regarding the morphology of these biomaterial particles and their size [21,22,23,24]. No authors have demonstrated whether a particular geometry of the spicules of antigenated and collagenated bovine bone provides the foundation for maximum response in terms of matrix deposition by osteoblasts. Previous works on hydroxyapatite have also demonstrated considerable effectiveness in terms of newly formed bone [25,26,27,28,29,30,31].

Some authors have published in vitro studies in which the effectiveness of materials that are commonly used in other medical fields was evaluated. In particular, the in vitro studies on the efficacy of raloxifene carried out by Memè et al. [32,33] demonstrated how, at least in vitro, it could be interesting to take advantage of the properties of some substances and combine them covalently with some biomaterials. Other authors have also published about raloxifene biomaterial devices [34]. In another work [35] concerning the different expressions of osteopontin, osteocalcin, and OB-cadherin in synthetic-nanohydroxyapatite-cultured vs. bovine-hydroxyapatite-cultured osteoblastic-like cells, the authors did not notice any statistically significant differences between the two materials, with both appearing to show equal expressions of these proteins. Campanella et al. [36] demonstrated the effectiveness of different biomaterials used in maxillary sinus lift; in particular, the materials used proved to be very similar in effectiveness but different in the percentage of material still available in situ after many months. This fact has never been given particular importance and clinical confirmation. In 2019, the studies by Nguyen et al. [37,38] changed the focus of many researchers’ attention; they highlighted how the inhibition of the periosteum that is in contact with the vestibular wall of a postextraction socket is able to ensure the filling of the same socket with newly formed bone. There are only in vitro studies that have demonstrated the effectiveness of static magnetic fields generated by a rubber dam [39], but these studies could open a new avenue for bone regeneration.

Another interesting way to use Ha is printed hydroxyapatite scaffolds; they could be used as a substitute for nanohydroxyapatite biomaterials used in clinical case reports. These 3D-printed hydroxyapatite scaffolds can be designed with a variety of pore sizes and architectures, which can be optimized to promote bone regeneration. Additionally, 3D-printed scaffolds can be customized to fit the specific needs of each patient [40,41].

Previous studies have unequivocally demonstrated that, at present, there is no biomaterial capable of being designated as the best in terms of osteoblast response; there is no scientific evidence suggesting that there is a biomaterial other than autologous bone, which is still defined today as the gold standard, capable of being osteoinductive and producing the best bone in terms of quality and quantity. Differences emerge regarding the quantity of residual biomaterial after 6/7/12 months in the bone cores performed in the grafted sites.

The purpose of this communication is to bring attention to the initial results obtained in a pilot case that used nanohydroxyapatite (NH) in socket preservation, where a large defect was restored and where a standard-diameter implant was placed to replace the missing tooth with good esthetics and function.

## 2. Materials and Methods

### 2.1. Objectives of Multicenter Study

A total of 100 patients requiring extraction of two adjacent teeth in the same quadrant will be recruited from 5 centers (10 patients per center). The multicenter study has the following objectives:

1. To evaluate and compare two postextraction sites treated with nano-HA in the posterior regions (test) and the control sites, thus involving 4 or 5 sites of the jaws, after 24 weeks of healing;
2. To determine the bone values of these sites in an evaluation during implant site preparation;
3. To determine whether the addition of nano-HA increases the amount of bone using a site evaluation;
4. To determine the bone quality of the sites based on a histological evaluation.

### 2.2. Study Population of Preliminary Study

The present preliminary study with 1-year of follow-up was a study performed in a private clinic in compliance with the principles of the 1964 Declaration of Helsinki for medical research protocols and ethics and its later amendments. The patient signed an informed consent explaining the use of MTG prior to the surgical procedure. The surgical procedure was performed in accordance with the postmarket clinical follow-up (PMCF) UE 2017/745. Participant was a healthy patient requiring one dental implant in upper 1.6. For the inclusion and the exclusion criteria, this study used the same criteria as the next multicentric study indicated in Section 2.3.

### 2.3. Study Population of Multicentric Study

The inclusion criteria are as follows:

- Age >18 years old;
- General good health (ASA I-II);
- Adequate oral hygiene (Full Mouth Plaque Score ≤ 20%, Full Mouth Bleeding Score ≤ 20%);
- Two adjacent teeth to be extracted in the same quadrant in 4 and 5 sites.

The exclusion criteria are as follows:

- Pregnant or within lactating period;
- Untreated periodontitis;
- Osteometabolic disease;
- Intravenous bisphosphonates therapy;
- History of chemotherapy or radiation therapy applied to the neck and head area;
- Frequent smoking habits (>15 cigarettes/per day);
- Absence of buccal bone plate;
- Patients with active local infection.

### 2.4. The Nano-Ha:NuvaBone

NuvaBONE (Overmed, Buccinasco, Milano, Italy) is an innovative line of synthetic bone graft substitutes that is based on nanostructured biomimetic hydroxyapatite for application in oral–maxillofacial surgery, orthopedics, traumatology, spine, and neurosurgery. NuvaBONE hydroxyapatite is a calcium phosphate biomaterial equivalent to the mineral matrix of human bone in morphology and composition [19]. Particularly, it has a hexagonal structure [20,21], and a stoichiometric Ca/P ratio of 1.67, which is identical to bone apatite [22] A major property of hydroxyapatite is its stability when compared with other calcium phosphates [23]. Thermodynamically, hydroxyapatite is the most stable calcium phosphate compound under physiological conditions, such as temperature, pH, and the composition of body fluids [24]. NuvaBONE biomaterials are manufactured through a patented process for the synthesis of new high-quality hydroxyapatite nanoparticles with extremely high purity and crystallinity to be used as biocompatible nanomaterial for biomedical applications. NuvaBONE biomaterials are based on micrometric aggregates of hydroxyapatite nanoparticles with a typical particle size of <50 nm and a rod-like shape (typically 30–40 nm length, 5–10 nm width) [2], available in different formats, such as dense granules, porous chips, and injectable paste. Due to the similarity between nanohydroxyapatite and mineralized bone, NuvaBONE biomaterials have a high affinity to hard tissues, as they form chemical bonds with the host tissue, resulting in an improved biological performance [6]. The porous and interconnected structure of NuvaBONE hydroxyapatite offers optimal osteoconductive activity, promoting cell penetration and colonization, circulation of nutrients, and rapid vascularization, allowing its complete degradation by osteoclasts and a whole remodeling into new vital bone tissue through a physiological time period of 6 to 12 months.

### 2.5. Surgical Technique

After local anesthesia, teeth will be extracted gently, and the residual sockets will be carefully debrided from all granulation tissue, rinsed, disinfected with sterile saline, and grafted. Randomization will indicate which socket will be grafted with pure NH and which will be grafted with NH associated with collagen gel.

The sockets will be packed to achieve a good contact between the bone and the biomaterial, and periapical rx will be taken at baseline and at 3 and 6 months.

At 6 months, implants will be inserted, and biopsies will be taken to assess the degree of regeneration induced by the two biomaterials. The ISQ of the implants will also be measured on the day of surgery and at 4 months after exposure. If the ISQ is higher than 65 at the time of insertion, a healing abutment will be placed as a one-time abutment. The implants will be followed and evaluated for up to 18 months, with periapical rx taken at 6/12/18 months.

Histological analysis and stability of the regenerated bone will be evaluated as well.

### 2.6. Case Presentation. Nano-HA Applied to Socket Preservation Procedures

**Figure 1:**
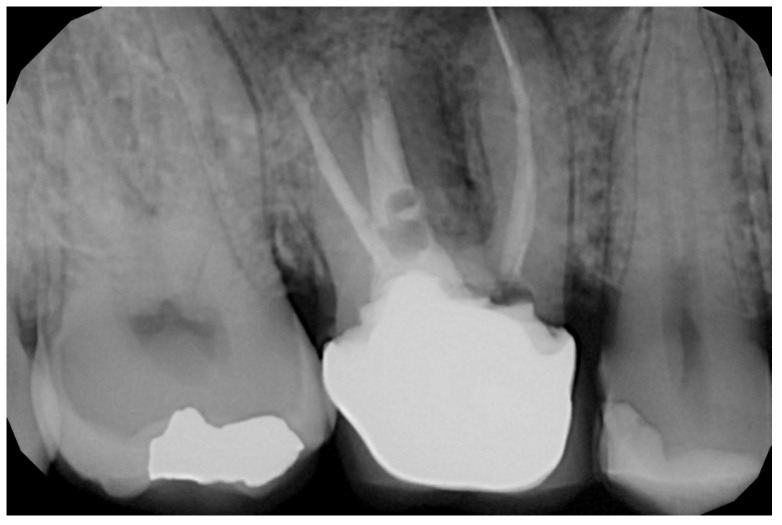
Hopeless upper first molar.

**Figure 2:**
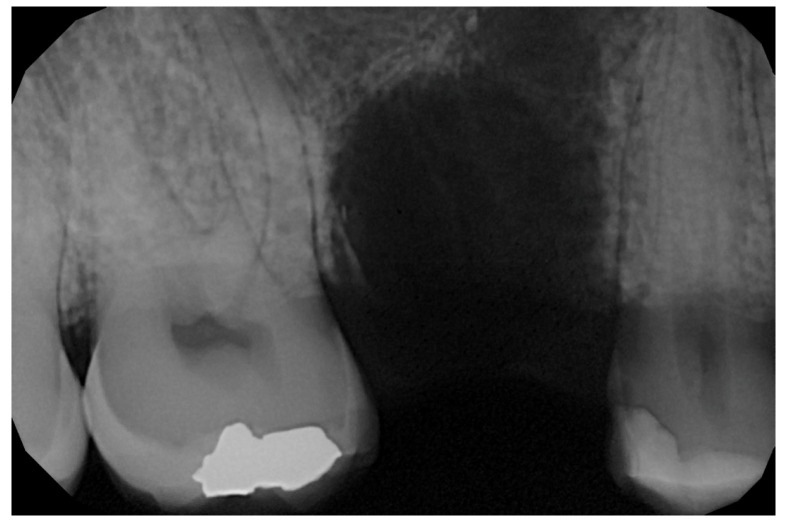
Residual defect after extraction.

**Figure 3:**
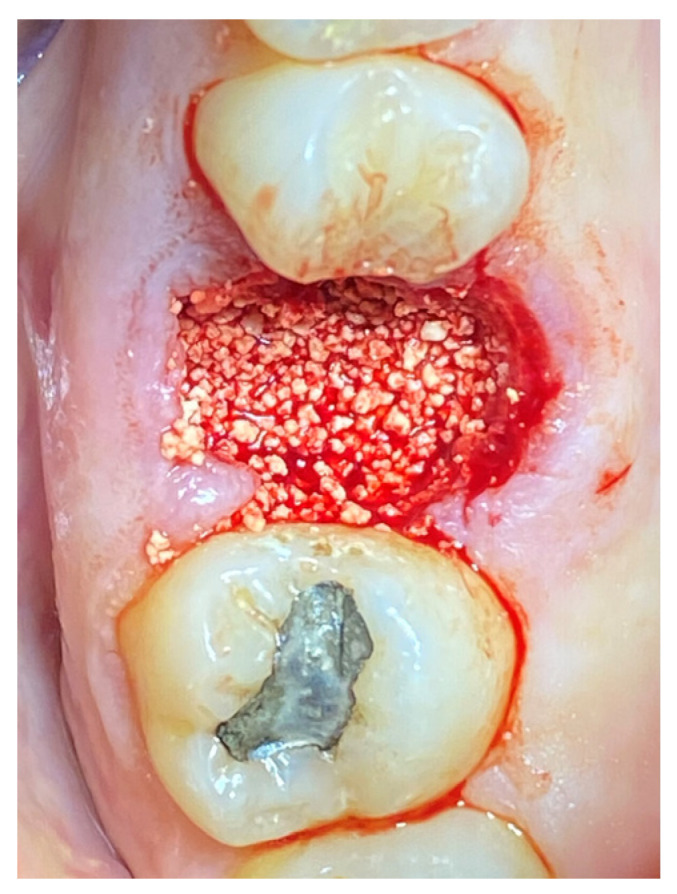
Graft in place.

**Figure 4:**
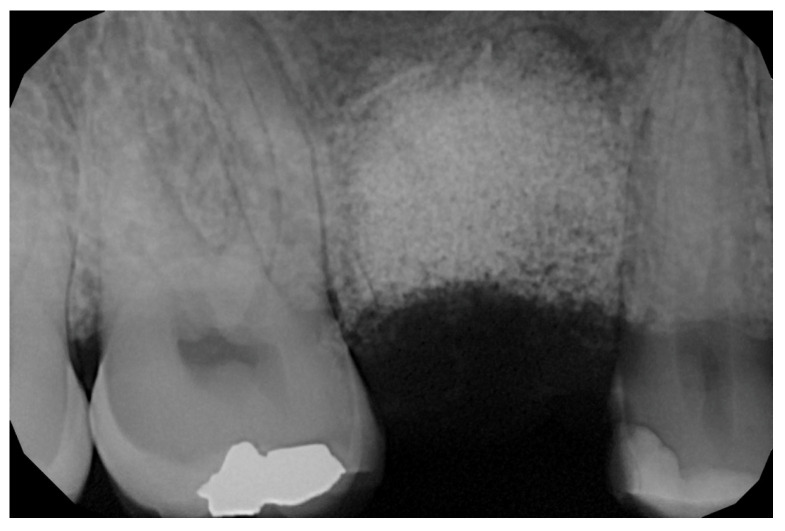
RX of the grafted site at baseline.

**Figure 5:**
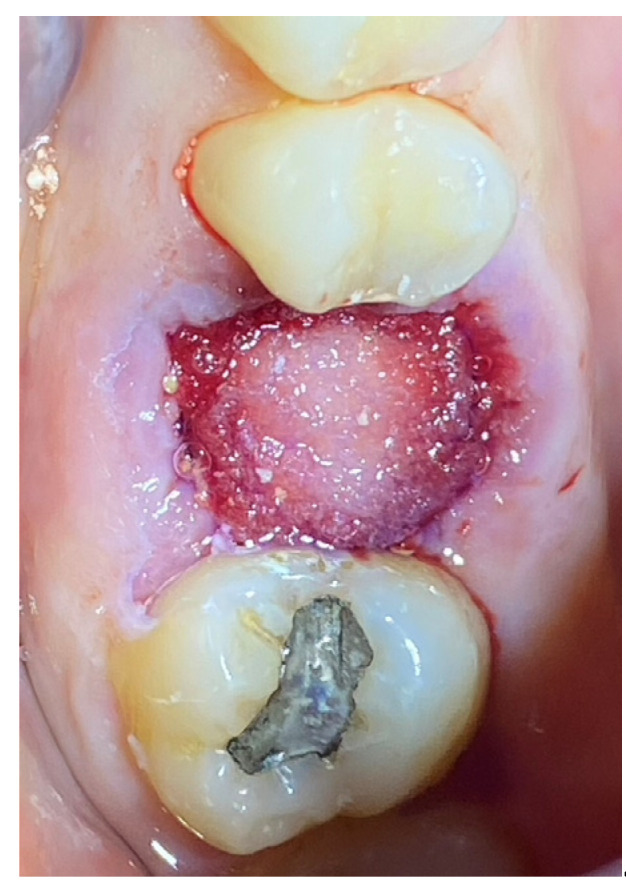
Socket sealed.

**Figure 6:**
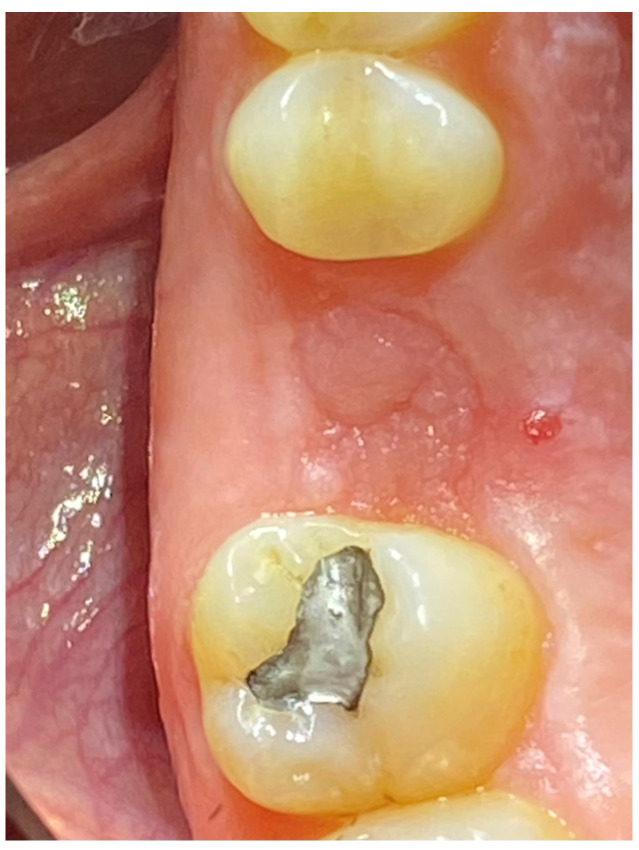
Healing at 6 months.

**Figure 7:**
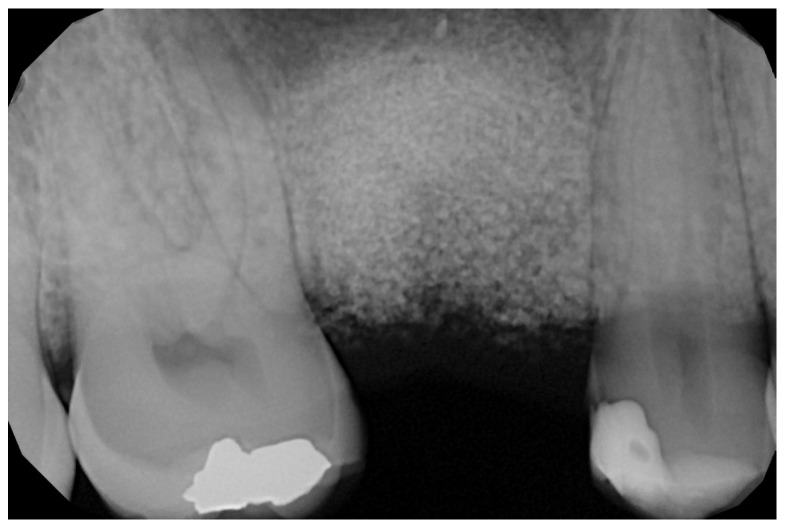
Grafted site at 6 months.

**Figure 8:**
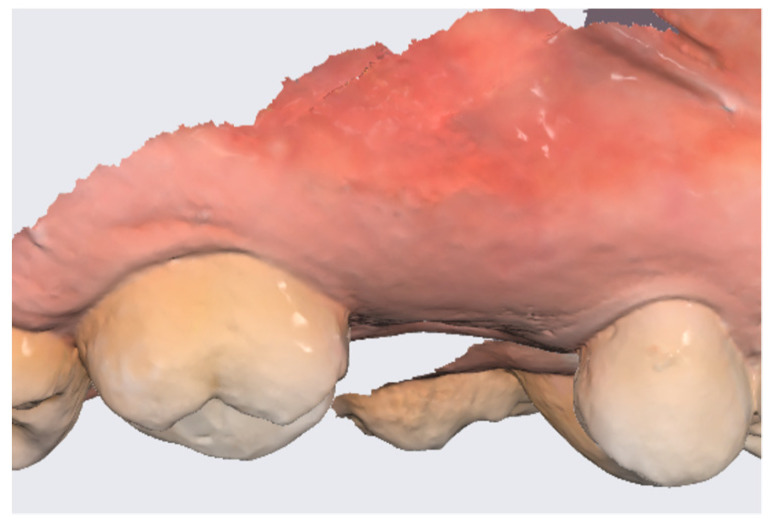
Lateral scan.

**Figure 9:**
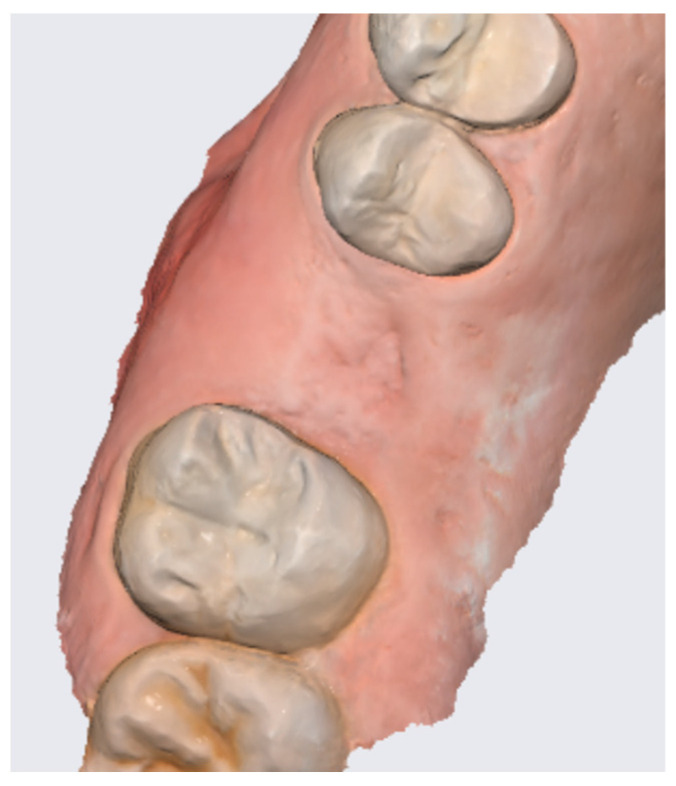
Occlusal scan.

In the pilot study, a 46-year-old man in good health presented to our clinic with a broken upper molar (Figure 1). The roots were very large, and after extraction, the residual defect did not allow the simultaneous placement of an immediate implant (Figure 2). The alveolar socket was debrided, disinfected, and grafted with NanoHA (Sphera, Overmend, Buccinasco, Italy); the socket was filled completely with NHA that was mixed with the patient’s own blood (Figure 3 and Figure 4). The procedure was completed by placing a fibrine sponge on top of the graft and by covering the area with octyl butyl cyanoacrylate (PeriAcryl, Bioteck, Arcugnano, Italy). Healing was uneventful (Figure 5, Figure 6 and Figure 7). At 6 months after grafting, the site remained stable, not showing any kind of volumetric loss. A scan of the area showed excellent maintenance of the 3D volume (Figure 8 and Figure 9).

**Figure 10:**
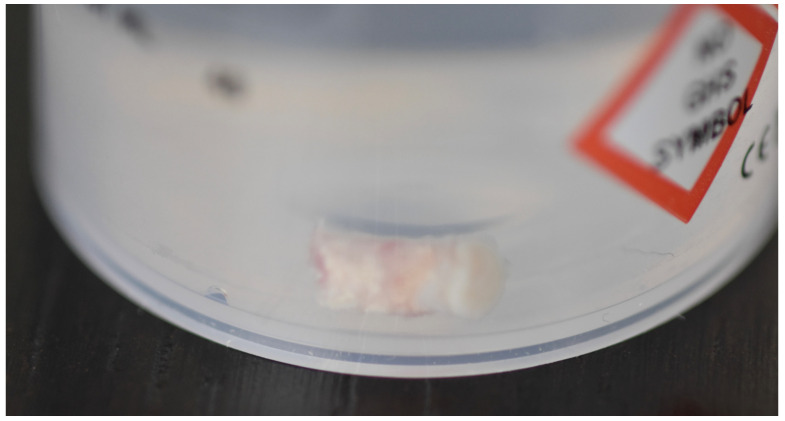
Biopsy taken from the site.

At the 12-month mark, the procedure to insert an implant was planned. The implant (Overmed, Buccinasco, Italy) was placed at the bone level after harvesting a core of the bone and soft tissue using a 4 mm (inner portion) trephine. The biopsy was sent to the Università Politecnica delle Marche (Ancona, Italy) for evaluation (Figure 10). The tissue samples were fixed in 10% paraformaldehyde for 48 h at 4 °C, washed in phosphate buffer with a pH of 7.4, and decalcified using Biodec R (Bio-Optica Milano S.p.A., Milano, Italy) for 6 h. The samples were then washed in PBS 1X and dehydrated by increasing the alcohol grade and xylene before paraffin embedding. Five-micrometer-thick tissue sections were cut, deparaffinized, and rehydrated using xylene and a graded series of ethyl alcohols. For histological analysis, the tissues were stained with hematoxylin and eosin and observed using a Nikon Eclipse 600 Light Microscope. The Fiji software 2.14.0/1.54f accessed in 7 July 2023 was used to reconstruct the full images via the plugin MosaicJ (https://imagej.net/software/fiji/downloads accessed on 30 Septembrer 2023) by acquiring pictures at 4× magnification.

## 3. Results

**Figure 11:**
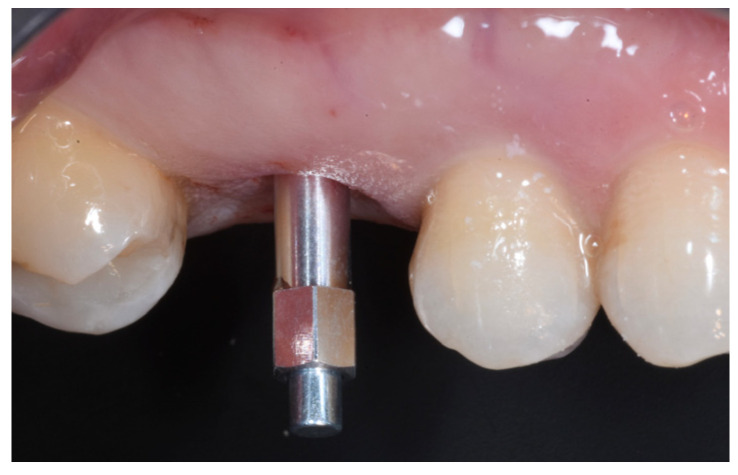
Implant testing based on the ISQ.

**Figure 12:**
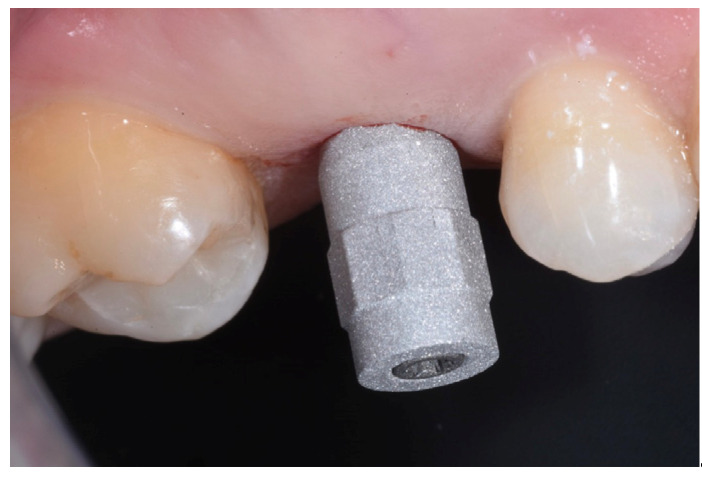
Scanbody in place.

**Figure 13:**
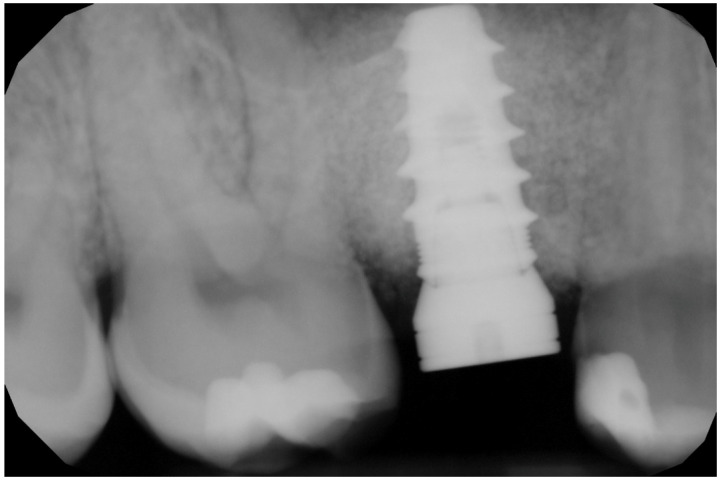
Healing abutment in place.

The ISQ values of the implant were 62, 62 (vestibular measurement, palatal measurement); therefore, a healing abutment was connected after taking a digital impression of the implant (Figure 11, Figure 12 and Figure 13).

**Figure 14:**
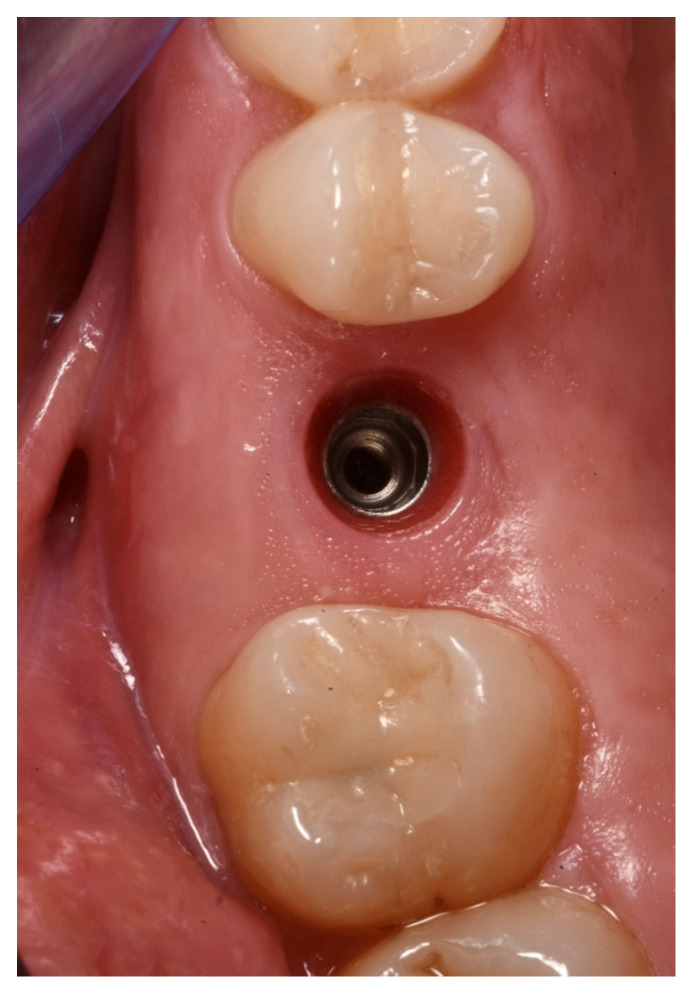
Soft tissue conditioning.

**Figure 15:**
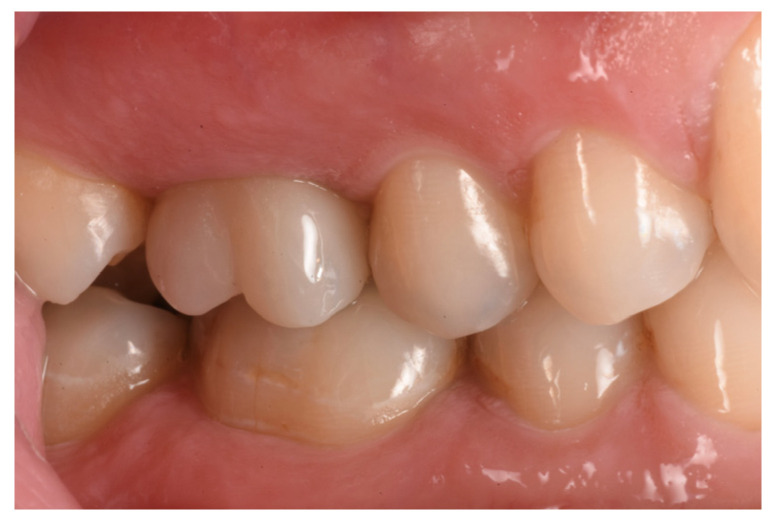
Final restoration in place.

**Figure 16:**
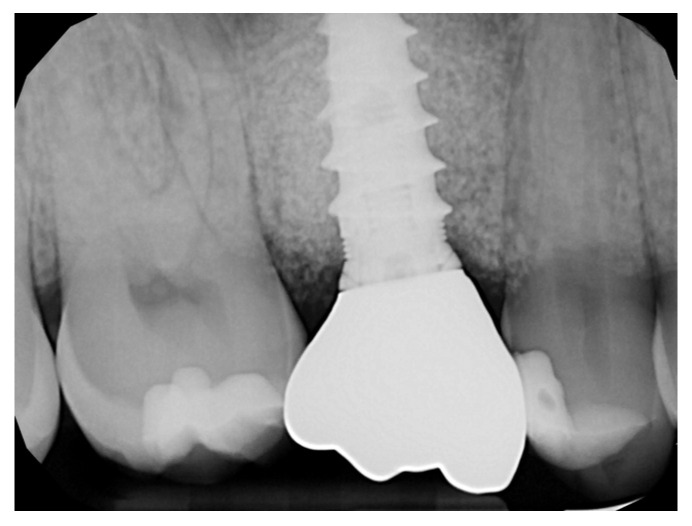
Rx of the final restoration.

The healing abutment led to a very healthy peri-implant tissue (Figure 14), and the ISQ values improved from an initial 61, 61 to 81, 81 (vestibular measurement, palatal measurement) on the day when the final restoration was delivered (Figure 15 and Figure 16).

**Figure 17:**
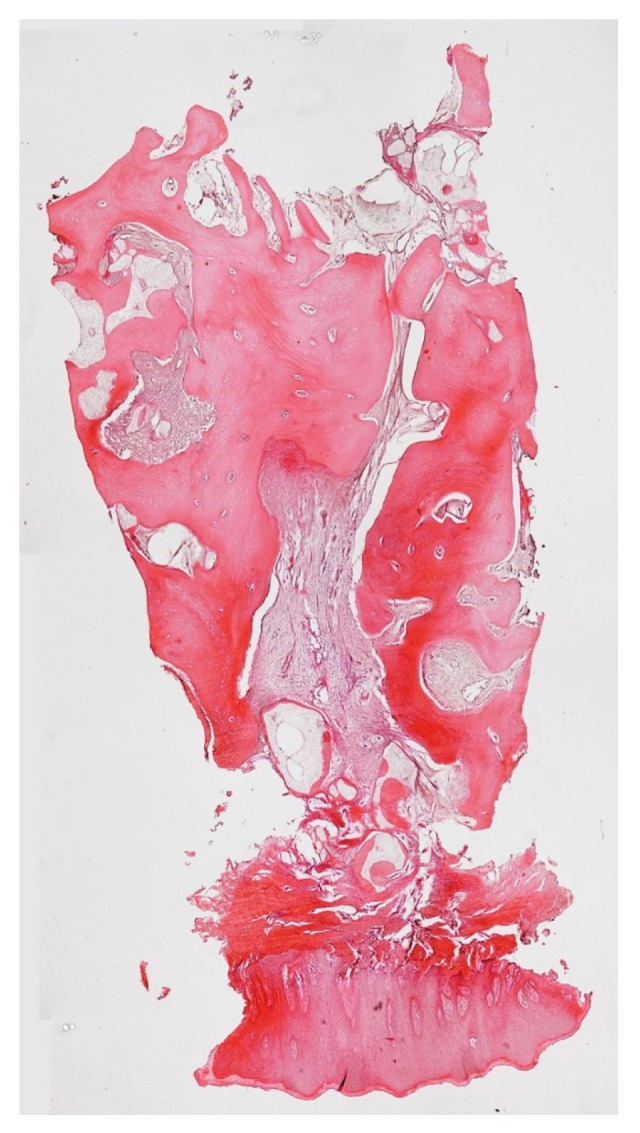
Biopsy during implant site preparation.

**Figure 18:**
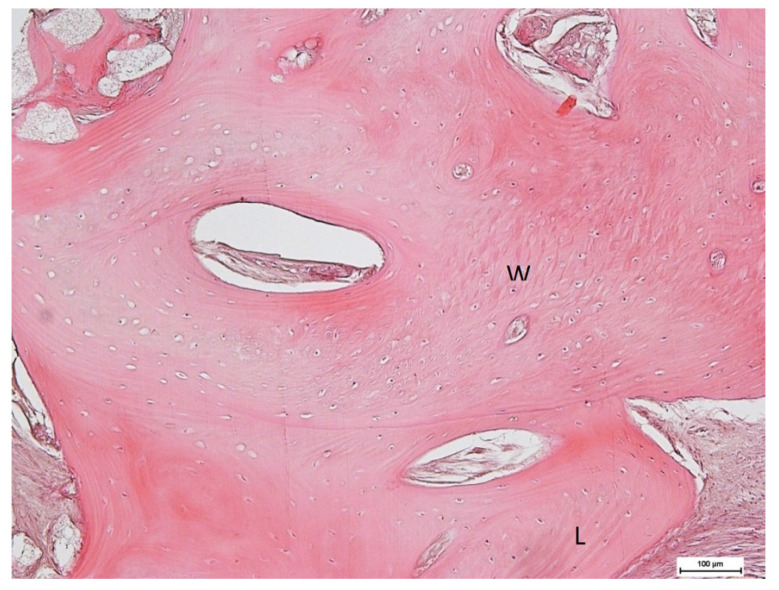
Woven and lamellar bone maturation.

Histological observation (Figure 17 and Figure 18) of the whole section showed a large area with bone tissue, connective tissue, and epithelium. At higher magnification, both woven bone and lamellar bone were evident in several areas, and new bone formation was evident. The final restoration data and radiograph showed how, in this case, NH was effective in facilitating ridge preservation and bone regeneration, prompting the idea that this promising result could be confirmed in a larger group of patients who could benefit from the use of this biomaterial.

## 4. Discussion

Today, the use of biomaterials in the clinical practice of dentists is considered a routine practice. The dream of dentists is undoubtedly to have a “magic potion” capable of regenerating large volumes of bone and perhaps even accelerating healing times. Unfortunately, current scientific evidence highlights that this is not yet realistic, and the majority of biomaterials work in exactly the same way, that is, they provide an optimal scaffold to give blood clots the possibility of proceeding with optimal healing via primary intervention. Therefore, studies that take into consideration the dentin and pulp of extracted teeth subjected to decalcification to fill postextraction sites are not interesting; the shredded dentin continues to progress as part of the bone and presents a cost-free autologous material [42,43,44,45]. Further, in this regard, researchers [46,47,48,49,50,51,52] have paid great attention to platelet concentrates and the different ways of preparing them. The results obtained using platelet concentrates are by far the best, especially for soft tissues and less so for bone. The nonspecific chemotactic process generated by the degranulation of platelet alpha granules enhances the regenerative capacity of both soft and hard tissues. However, even though it is beneficial to use platelet concentrates, one must not ignore the inconvenience of having to take a quantity of blood from patients, a procedure that is not yet permitted for dentists in some countries. Therefore, there remains a need to carefully evaluate the results in terms of bone quality after the application of these biomaterials.

The histological specimen of a nano-HA grafted area showed complete graft replacement with little remnants of the nano-HA graft material. It is known that the residual presence of biomaterial in newly formed bone alters not only the quantity but also the quality of the regenerated bone and the consequent possibility of inserting an osseointegrated implant in that site. Although more in-depth studies are necessary, we can assume that this result is due to the peculiar characteristics of Nano-Ha; in particular, the size of the particles, and the nanoporosity favored faster reabsorption and, consequently, left room for a greater quantity of mature bone. The presence of mature bone would explain the high ISQ value found at the time of implant insertion; the value also increased at the time of prosthesis. The increase in the ISQ value is explained by the completion of the osteointegration of the implant, which occurred precisely due to a normal vascular organization of the newly regenerated bone. This article presented a single case report, which means that the findings cannot be generalized to other patients. We used nanohydroxyapatite biomaterial without a control site, so we cannot conclude whether the results would be the same with or without biomaterial.

For these reasons, a multicenter study is very important to answer many questions; however, in our pilot case, the quality and quantity of bone proved to be of a high level after an observation of the material undergoing reabsorption and replacement.

## 5. Conclusions

This case report showed the successful clinical use of a nano-HA graft for socket preservation. We want to indicate three good results on which we can focus our attention: *high bone quality* based on histological evaluation, *high resonance frequency* values based on the Osstell scale, and *low residual material* in the histological samples. There are many biomaterials on the market and testing a new biomaterial could be expensive and useless; the preliminary study served precisely to understand whether this nano-Ha possessed characteristics that would encourage a multicenter study.

The next multicentric study will be coordinated by the Polytechnic University of Marche and Dr Roberto Rossi.

## Data Availability

Data are contained within the article.
